# The effect of commitment-making on weight loss and behaviour change in adults with obesity/overweight; a systematic review

**DOI:** 10.1186/s12889-019-7185-3

**Published:** 2019-06-24

**Authors:** Nia Coupe, Sarah Peters, Sarah Rhodes, Sarah Cotterill

**Affiliations:** 1 Manchester Centre for Health Psychology, School of Health Sciences, The University of Manchester, M13 9PL, Manchester, UK; 2 Centre for Biostatistics, School of Health Sciences, The University of Manchester, Manchester, UK

**Keywords:** Obesity, Behaviour change techniques, Commitment, Behavioural contract, Weight loss

## Abstract

**Background:**

Adherence to weight loss interventions is crucial to successful outcomes, yet little is known about how best to improve it. This suggests a need for developing and improving adherence strategies, such as formal commitments. This review aims to identify the effect of including a commitment device alongside lifestyle interventions on weight loss, and identify the most appropriate delivery mechanisms and target behaviours.

**Methods:**

We searched five databases and hand-searched reference lists for trials of behavioural interventions to achieve weight loss among adults with excess weight or obesity. Interventions incorporating commitment devices were included in a narrative review and meta-analysis where appropriate. Commitment devices with financial incentives were excluded.

**Results:**

Of 2675 unique studies, ten met the inclusion criteria. Data from three randomised trials including 409 participants suggests that commitment interventions increases short-term weight loss by a mean of 1.5 kg (95% CI: 0.7, 2.4). Data from two randomised trials including 302 patients suggests that benefits were sustained at 12 months (mean difference 1.7 kg; 95% CI: 0.0, 3.4). Commitment devices appeared most successful when made publicly, and targeting diet rather than physical activity.

**Conclusions:**

Using commitment devices, such as behavioural contracts, as part of a weight loss intervention may be useful in improving weight loss outcomes and dietary changes, at least in the short-term. However, evidence is limited and of variable quality so results must be interpreted with caution. Poor reporting of intervention details may have limited the number of identified studies. More rigorous methodology and longer term follow-ups are required to determine the effectiveness of behavioural contracts given their potential for use in public health interventions.

## Background

The global prevalence of obesity more than doubled between 1980 and 2014, with 13% of adults classified as having obesity, and 39% as overweight in 2015 [1]. Obesity increases people’s risk of developing type 2 diabetes [2], stroke [3], cardiovascular disease [4], certain cancers [5] and all-cause mortality [6]. There is clear evidence that people with a body mass index (BMI) ≥ 25 kg/m^2^ have increased healthcare costs [7]; medical costs of weight-related health conditions are estimated at £6 billion in the UK [8]. Obesity is a major public health issue. However, such effects can be reversed, thus decreasing the associated medical costs; intentional weight loss can decrease the risks of mortality [9], cardiovascular disease [10], and type 2 diabetes [11]. The most effective interventions for weight loss are those which focus on both dietary and physical activity changes [12, 13], yet outcomes remain suboptimal due to poor adherence to such interventions [14]. As such, it has been suggested that the inclusion of dietary adherence strategies may be more important than diet type [15].

The inclusion of a commitment device may be one such approach to improve adherence to lifestyle interventions. ‘Commitment device’ is an umbrella term for any technique that helps people commit to performing a particular behaviour, or achieving a particular outcome. They include behavioural contracts, pledges, verbal agreements/commitments, and can be categorised into two broad categories; ‘soft’ and ‘hard’. Hard commitments have “real economic penalties for failure, or rewards for success” [16], such as contingency contracts, whereby the individual deposits money which is only returned on attainment of the goal to which they have committed. Soft commitments do not have any incentives or rewards attached, but tend to rely on social consequences such as making a commitment publicly [17].

A systematic review of health promotion contracts (including soft and hard commitments) between patients and health professionals identified that whilst there is limited evidence that they can improve adherence, there were not enough large and high quality RCTs to support their routine use [18]. Three of the 30 trials included addressed weight loss, but none reported adherence outcomes, therefore supplying little evidence about the effect of commitments on adherence. However, short-term weight loss outcomes were higher in the contract groups than control groups in two of the three studies, suggesting that commitment making warrants further exploration. Another systematic review focusing on the effects of financial (hard) contingency contracts [19] concluded that they were effective in the short-term, but the behaviour did not last beyond the intervention period. This short-term effect of incentives on behaviour change for weight loss is supported further by another review which included different types of incentives, including cash payment and premium reductions, and concluded the same [20]. These studies suggest that the effect only lasts whilst participants are receiving the incentive, and therefore may not be suitable for weight loss maintenance.

Soft commitment devices have shown promising results in pro-environmental behaviour change research and results suggest that commitment-making may have longer term effects than other behaviour change techniques (BCTs), such as incentives [21]. This is important given that many regain weight lost following the intervention period [22]. Though historically it was postulated that extrinsic motivation (e.g. incentives) could undermine internal motivation [23], more recent studies have shown this is not the case in relation to educational attainment [24] or indeed weight loss [25]. Incentives may be more suited to increasing the number of tasks completed, and to short-term effects, supported by the short-term effects previously discussed [19, 20]. As contingency contracts require individuals to self-fund deposits, they are an unsuitable approach for low socio-economic populations. Other approaches which pay individuals to reach their goals may be more suited to this population, but are not financially sustainable, and may be viewed as unethical by the public [26].

In addition to being more acceptable, soft commitments may be a more economically prudent approach to health behaviour change [27]. A recent review of different types of online commitments concluded that though financial incentive groups self-reported more weight loss, commitment contracts assisted weight loss across all groups, suggesting that they remain effective without financial incentives, though to a lesser extent [28]. Soft commitment devices are brief, simple to incorporate, cost-effective and may be easy to understand for those with low health literacy, it is important to establish if they can promote behaviour change and maintenance in relation to weight loss. The aims of this review therefore were to 1) determine the short and longer term effect of soft commitment devices targeting physical activity and/or diet on weight loss outcomes amongst adults with obesity or excess weight 2) determine if commitment devices are more effective for adherence to certain lifestyle behaviours 3) identify which delivery elements and behaviour change techniques (BCTs) are associated with positive outcomes.

## Methods

A protocol for this review is available online at www.crd.york.ac.uk/PROSPERO (ID number CRD42018102506).

### Study inclusion criteria

#### Population

Studies were included if they recruited adults ≥18, with a body mass index (BMI) of ≥25 [29], or if amount overweight or weight loss goal ≥15lbs (as reported by authors), or percentage overweight ≥10%. A scoping review identified potential studies that were conducted before BMI reporting became commonplace, therefore we did not limit to BMI only given this would potentially exclude some relevant studies.

#### Interventions

Studies were included if the intervention comprised a soft commitment device accompanying a behavioural lifestyle intervention targeting diet and/or physical activity, which is the recommended course of action for weight loss [29]. We defined a soft commitment device here as:

*‘A verbal or written commitment to adhere to a health behaviour (e.g. diet/exercise), and/or to achieve a desired outcome (e.g. weight loss). The commitment must be witnessed by another (in person or online), and can take place at the beginning or at multiple times throughout the intervention. There should be no material incentive or reward attached to the commitment’*

We provide our own definition here, given that some forms of commitment devices identified in our scoping review were based on commitment to achieving a certain outcome (rather than performing a behaviour), which would not be included if we had exclusively used the definition of the BCT ‘commitment’ from the Behaviour Change Technique Taxonomy version 1 (BCTTv1) [30]. Interventions involving financial incentives relating to the commitment goals were excluded, because this review aimed to determine the effect of the commitment itself rather than any financial reward. Those that included a course fee or deposit not relating to the contract goals (e.g. for attendance) were included as they were not contingent on the contract behaviour or outcomes, and therefore were not identified as an incentive. Interventions that included medical interventions (e.g. bariatric surgery) or weight loss medications were excluded.

#### Comparisons

Control groups included no intervention, or similar interventions without the commitment device (aims 1 & 2). Studies with controls which included a different commitment device, or the same commitment device with different sub-components, were also included (aim 3).

#### Outcome measures

The primary outcome was change in weight (kg) or BMI, given the associated health and cost benefits of weight loss in this population. Secondary outcomes were behaviour change, including self-reported or objective measures, given that we were interested in how/if commitment devices affect behaviour.

#### Setting

All settings were included.

#### Study design

Study design included randomised or non-randomised controlled trials.

### Search strategy

Five databases were identified, informed by reviews in this area (e.g. [31]), and through suggestions from a library based systematic review specialist. The following five databases were searched from inception to March 2018: Medline, Embase, CINHAL, PsycInfo and Web of Knowledge. A scoping review identified keywords and associated MESH terms around the four key topics; obesity/overweight, weight loss, lifestyle interventions, and commitment BCTs (see Appendix 1). Further studies were sought through hand searching reference lists of identified studies. The search was conducted in March 2018, and was restricted to English language only, with no restrictions on date.

### Selection criteria

Titles and abstracts were screened against the study inclusion criteria by the first author. Full texts were obtained for those which met the criteria, or where the abstract contained insufficient information to exclude. Full texts were screened, and 15% (*n* = 22) were independently double-screened, with 100% agreement. Insufficient information was identified in nine papers, and authors contacted for clarification, with seven responses.

### Data extraction

The ERC data collection form [32] was used to record exclusion reasons and extract data from the included studies, including population and setting, methods, participants, intervention details, outcomes and results.

### Quality assessment

The Cochrane Collaboration’s risk-of-bias tool [33] was used to assess methodological quality across six domains (sequence generation, allocation concealment, blinding, incomplete outcome data, selective reporting and other), rated high, low and unclear. Results were reported across individual studies and by domain. Two authors rated bias for each paper independently, and any discrepancies were resolved through discussion among the authors to reach a consensus.

The Behaviour Change Technique Taxonomy v.1. [30] was used to code the behaviour change techniques (BCTs) from all interventions. The first author, and another researcher with experience of the taxonomy, who is not an author of this paper, double coded the BCTs. We calculated inter-rater reliability using PABAK (prevalence-adjusted-bias-adjusted-kappa) [34], with very good agreement (PABAK = .93). We chose PABAK over Cohen’s kappa given it is better suited to dealing with coding BCTs, where there is a high proportion of negative responses [35]. Discrepancies were resolved through discussion.

### Data synthesis

As we expected data to be heterogeneous, a random effects analysis was planned as per the Cochrane Handbook guidance [33]. We also conducted a sensitivity analysis switching to fixed effects, and our conclusions remained the same. We had appropriate data (mean weight loss in kg) from four studies, three of which were suitable for inclusion in a random effects meta-analysis. One study did not report standard deviations for weight loss [36], and the standard deviation (SD) was calculated following the steps laid out in the Cochrane handbook for systematic reviews of interventions [33].

Narrative synthesis was used to describe the remaining studies, which is suitable for describing heterogeneous studies [37].

## Results

**Fig. 1:**
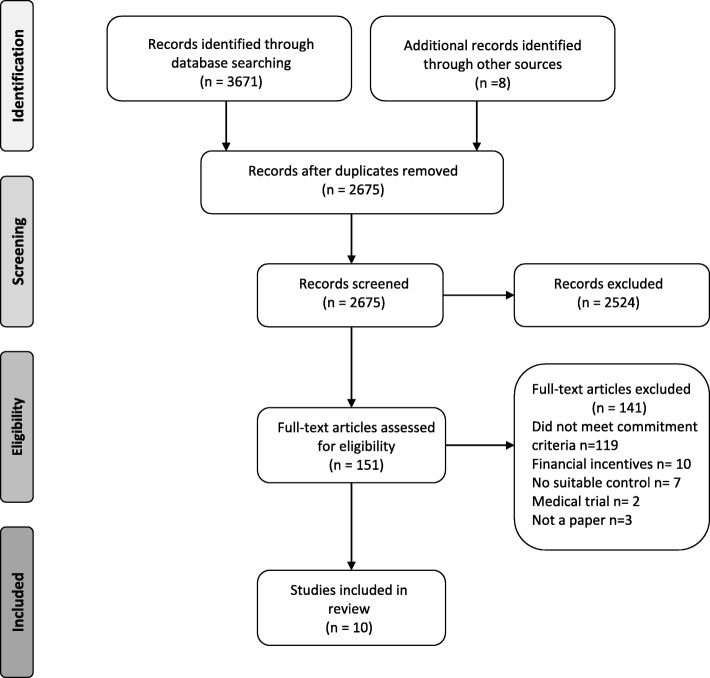
PRISMA flow diagram of search procedure for narrative review and meta-analysis

Of 3671 unique studies identified through database searches, ten studies were included in the final review (Fig. 1).

### Study characteristics

Studies were published between 1980 and 2017 (five in 1980s, one in 90s and four since 2010) in the USA (*n* = 7), Denmark [38], India [39] and Japan [40], with a total of 1320 participants (see Table 1). Seven studies took place within community settings, two took place in the workplace [38, 40] and one home intervention [41]. Six studies reported mean age, ranging from 30 to 59 years [36, 38, 40–43]. All but one study [44] reported gender: of the 1214 participants, 1041 (86%) were female. Four studies exclusively recruited women [39, 41, 43, 45], one recruited only men [40]. In terms of commitment device type, eight studies reported the use of behavioural contracts (1.8 BCTTv1), one specified the use of verbal commitments (1.9) by male spouses of female participants in addition to the contracts [43], one described an online/app based pledge [38], and one reported a public commitment to an outcome (rather than a behaviour) [39]. Details regarding the content of the weight loss interventions were largely unavailable, but reported content was traditional weight management education focusing on dietary change (e.g. reducing calorie/fat content) and increasing physical activity (Appendix 2).

**Table 1:** Details of studies included in review

Study ID (country)	N (gender)	Weight status	Setting	Intervention format	Intervention length	Deliverer	Comparison	Deposit/ Fee
Balk-Møller 2017 ^a^ [38] (Denmark)	269 (92% female)	Mean waist circumference 92 cm	Workplace	Individual / Workplace team	38 weeks	Web and App	D/PA + pledge vs NTC	None
Black ^b^ 1983 [43](USA)	36 (females)	37% mean overweight**	Community	Couple / Individual	10 weeks	HCP + DS	D/PA + BC with varying husband involvement	$11 deposit refunded on attendance
Clifford 1991 ^a^ [36] (USA)	48 (completed 17 female 17 male)	84% had BMI ≥ 25	Community	Group + individual /peer support	52 weeks	HCP/ YMCA director	D/PA + BC vs NTC	$50 NR deposit $195 course fee
Craighead 1989^a^ [45] (USA)	62 Female	15-45lbs overweight	Community	Group information + supervised/contracted exercise	12 weeks	DS	D/PA + contract vs contract only	$10 deposit refunded at end of treatment
Dubbert ^b^ 1984 [46] (USA)	62 (48 female,14 male)	Min 15lbs, max 100% overweight*	Community	Group + Couple/Group + Individual	19 weeks	HCP	D/PA + spouse BC vs D/PA, proximal or distal goals.	$65 deposit ($15 refundable) Cost for couples.
Franzini 1980^a^ [42] (USA)	76 (70 female, 6 male)	37% mean overweight** (10.4–87.7%)	Community	Group	11 weeks	HCP + GS	D/PA + BC vs D/PA vs WLC	£5 cost for materials
Kegler ^a^ 2016 [41] (USA)	349 (female only)	BMI 38.3 (mean)	Home	Individual + telephone support./ 3 mail information	16 weeks	Non-HCP	D/PA + BC vs IO	None
Maruyama 2010 ^a^ [40] (Japan)	101 (male only)	BMI 25.8 (mean)	Workplace	Individual + Web support	16 weeks	HCP	D/PA + BC vs NTC	None
Nyer ^a,b^ 2010 [39] (India)	211 (female only)	15-20lbs* overweight (20lbs max)	Community	Group	16 weeks	HCP	D/PA + BC conditions vs D/PA	Not stated
Ureda ^b^ 1980 [44] (USA)	106 (not reported)	34.5 lbs. (median)	Community	Group	4 weeks	Not reported	D/PA + BCW vs D/PA + BC	None

^a^commitment vs control study, ^b^commitment vs different contract study. HCP Healthcare Professional, GS Graduate student, DS Doctoral student, D Dietary intervention, PA Physical activity intervention, WLC Waiting list control, NTC No treatment control, IO Information only, BCW Behavioural contract witnessing BC Behavioural contract

Studies were inconsistent in relation to the categorisation and reporting of participants’ weight status at baseline, possibly due to the age of the studies. Participants were included in the studies based on either BMI, amount overweight (% or lbs), or weight loss goal. Six studies reported actual weight ranges of participants, one reported average waist circumference, whilst three reported the inclusion criteria only (see Table 1).

### Study designs

Study design varied in terms of what was offered to the comparison groups (Table 2). These different study designs are not easily comparable, but the results answer different aims and are presented in relation to these.

**Table 2:** Studies included in review by design type

Study design	Study
1. Commitment intervention versus minimal contact/ assessment only	Clifford 1991 [36], Balk-Møller 2017 [38], Maruyama 2010 [40], Kegler 2016 [41]
2. Commitment intervention versus same intervention without commitment condition and variation of commitment condition	Nyer 2010 [39]
3. Commitment intervention versus minimal contact/ assessment, same intervention without commitment condition, and variation of commitment condition	Franzini 1980 [42]
4. Commitment intervention versus variation(s) of commitment condition only	Black 1983 [43], Craighead 1989 [45], Dubbert 1984 [46], Ureda 1980 [44]

### Primary outcome

#### Weight outcomes- commitment intervention

As seen in Table 2, four of the included studies reported interventions that included a commitment device as a major component of the intervention, compared with a minimal contact / assessment only control, and provided useful data in relation to weight, three of which were included in a meta-analysis.

**Fig. 2:**
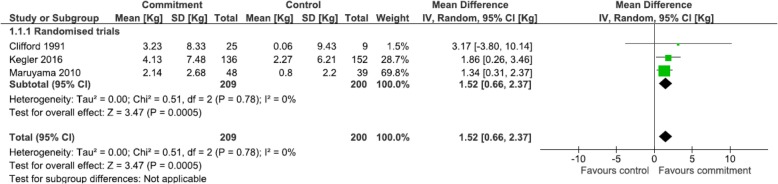
Meta-analysis of short term (≤6 months) weight loss

*Short-term weight outcomes for comparative commitment interventions (≤6 months)* When randomised studies that reported mean weight loss [36, 40, 41] were pooled in a meta-analysis, heterogeneity was low. There was a statistically significant difference in weight change between the commitment device (all included behavioural contracts) intervention and control groups, with the commitment device group having on average a 1.52 kg greater weight loss (95%CI: 0.66, 2.37) as seen in Fig. 2.

Though analysis in the fourth trial [38] had been adjusted for clustering, this was by municipality rather than by the unit of randomisation which was care home. In addition, we are only interested in the weight loss sub-group, yet there is no weight loss only control therefore the entire control group has been used. Given these issues, it was not included in the meta-analysis. When we compare the weight loss sub-group with the entire control group, weight losses were higher than those which have been included (− 2.36 kg, 95%CI: − 3.23, − 1.49).

Neither of the two studies [39, 42] that included a direct comparison of the same intervention with and without a commitment device reported actual weight lost, and therefore we were not able to pool the results. Nyer and Dellande [39] reported a significantly higher percentage of those in the public commitment group achieved their weight loss goal than those in the control group at two (97.06 vs 90.05, *p* < 0.01) four (96.59% vs 88.86%, *p* = 0.01) and six months (89.10 vs 81.42, *p* = .01). Franzini and Grimes [42] reported mean percent weight lost, and found that whilst both groups lost significantly more weight than the assessment only control (4.51, 3.73% vs 0.3%); there was no statistical difference between intervention groups at end of treatment (13 weeks).

**Fig. 3:**
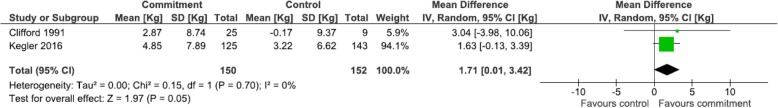
Meta-analysis of longer term (12 months) weight loss

*Long-term weight outcomes for comparative commitment interventions (12 months)* Two studies reported longer term outcomes [36, 41]. When pooled in a meta-analysis (Fig. 3), heterogeneity was low, and there was evidence of sustained weight loss with a 1.71 kg greater weight loss on average in the behavioural contract intervention groups compared to control (95% CI: 0.01–3.42).

#### Weight outcomes- commitment variations

As seen in Table 2, five of the included 10 studies compared variations of behavioural contracts, and three of these did this exclusively without a non-contract control [43, 44, 46]. Four studies focused on the involvement of others such as peers or healthcare professionals, either through publicity, attendance, or co-signing of contracts [41, 43, 44, 46]. Nyer and Dellande [39] compared two levels of public commitment groups which are discussed here. The fifth study included two behavioural contract groups in which we are interested; one alongside a group intervention and one alongside an educational booklet with minimal contact [45].

*Short-term weight outcomes for commitment device variations (≤6 months)* Results of co-signing or witnessing were mixed in relation to weight outcomes, and appear to be dependent upon who witnessed or co-signed the commitment device. Publicity of commitment was identified as important in one study, where percent weight loss goal achieved was increased at two (+ 4.71%, *p* = .04), four (+ 5.79%, *p* = .05) and six months (+ 8.36%, *p* = .01) by increasing the length of time they were public from 3 weeks to 16 weeks [37]. The authors state that the involvement of spouses (husbands of female participants) had no significant effect on short-term (3–6 months) weight outcomes of participants in two studies [43, 46], though no summary data was provided. Actual weight loss is not reported in one study [44], but authors state that the speed with which people lost weight significantly increased in the short-term (10–15 weeks) when behavioural contracts were co-signed by peers, relatives or friends. However, authors did not provide summary statistics, report numbers in each category, nor did it specify if they included spouses.

In Craighead & Blum’s [45] study, those contracting in a group setting lost significantly more weight than the contracting minimal contact group posttreatment (3.7 kg 95%CI: 3.39, 4.01 vs. 2.1 kg 95%CI: 1.69, 2.51).

*Long-term weight outcomes for commitment device variations (12 months)* Long-term weight outcomes were reported by two studies [43, 45]. There were mixed results regarding the involvement of spouses (husbands of female participants) on long-term weight outcomes in Black and Lantz’s [43] study. The husband absent group (no husband involvement, contract signed by counsellor) lost significantly more weight than husband not contracting group (passive involvement in intervention, contract signed by counsellor) at 12 month follow up (3.71 vs 1.09 kg, *p* < .05), but did not significantly differ from the amount of weight lost by the husband contracting group (2.43 kg) (full involvement of husband, contract signed by husband). These results suggest that male spouses of female participants should either be actively involved in the intervention and contract singing, or should not be involved at all (e.g. contract signed by intervention deliverer).

In Craighead and Blum’s [45] study comparing an exercise contract with or without a group intervention, results were similar at 12 months (2 kg, 95%CI: 1.62, 2.38 vs 1.9 kg, 95%CI: 1.40, 2.40), suggesting that behavioural contracts are only effective when used alongside a group intervention or ongoing support.

### Secondary outcomes – Behavioural outcomes and adherence

*Short-term (≤6 months) physical activity outcomes and contract adherence* Of the six studies comparing a commitment device intervention versus minimal contact, three reported physical activity outcomes, two of which used behavioural contracts and reported that the intervention did not increase self-reported or objective physical activity [40, 41] in the short-term (4–6 months). Clifford and colleagues reported a statistically significant effect in favour of the intervention group in relation to subjective measures of exercise adherence and objective measures of cardio-fitness at 6 months, though summary statistics were not provided [36]. The two studies that reported no effect on physical activity specifically included physical activity goals in their behavioural contracts (see Appendix 2 for further information regarding contracts within individual studies), suggesting that contracting to physical activity behaviour change is not effective. This is supported further as these two studies used objective measures (accelerometer/pedometers), whereas the exercise adherence measure used by Clifford et al. [36] was a subjective self-reported questionnaire, though the improved fitness levels do seem to corroborate the subjective measures. Clifford et al. [36] did not report the behavioural goal specified in the contract, but described a behavioural health change contract, so adherence to the actual contract behaviour is unclear. Two factors which may have affected outcomes was that study had a large fee attached ($195) and a non-refundable deposit of $50, which may have increased people’s commitment beyond that of the other two studies.

Of the studies comparing different commitment conditions, two reported adherence data, and found no difference between groups in relation to short-term exercise adherence [46] or fitness levels [45]. The specific behaviours specified in the behavioural contract used in Dubbert and Wilson’s study [46] were not reported, so the effect of the contract on adherence is unclear. As previously stated, the contracts in Craighead and Blum’s [45] study were exercise specific, suggesting that exercise contracts do not improve fitness outcomes.

*Long-term (12 months) physical activity outcomes and contract adherence* Two of the ten studies reported longer term outcomes, with no significant difference between groups reported in self-reported physical activity [41] or fitness outcomes [45].

*Short-term (≤6 months) dietary change and contract adherence* Two studies reported dietary adherence outcomes, and both reported that the behavioural contract interventions facilitated short-term (4–6 months) dietary change. The intervention group in Kegler et al’s [41] study consumed 205 fewer calories per day than the minimal contact control group (− 274 kcal 95% CI: − 371.98, − 176.02 vs. -69 kcal 95% CI: − 163.43, 25.43). The intervention group in Maruyama et al’s [40] study increased the number of healthy food groups consumed more than controls (mean difference 2.3, 95% CI: 1.0,3.7) and decreased the number of unhealthy food groups as specified in the contracts more than controls (mean difference 2.7, 95% CI: 0.9,4.5).

Of the studies comparing commitment device variations, one study reported dietary outcomes and found no significant difference in eating inventories between groups in the short-term [45].

*Long-term (12 months) dietary change and contract adherence* Kegler et al. [41] included longer term outcomes, and reported a larger decrease in the intervention arm (− 195 kcal 95%CI: − 288.79, − 101.21 vs. -76 kcal 95%CI: − 171.72, 19.72). Though self-reported, these results suggest that behavioural contracts can facilitate longer-term dietary change when behaviour specified in the contract is specific in relation to dietary behaviour to be performed.

Craighead and Blum [45] also reported longer term outcomes, and found no significant difference in eating inventories between groups. As previously stated, the behavioural contracts were exercise specific, therefore this does not provide evidence against their use for changing dietary behaviours.

### Delivery elements across all studies

#### Behaviour change techniques

Three studies [36, 40, 41] compared behavioural contract groups with minimal contact/no treatment controls only, so it is difficult to infer meaningful results regarding the contracts themselves, rather than the intervention as a whole. However, as they were all effective in terms of weight outcomes, we have identified the four common BCTs alongside which the contracts were delivered, which were: goal setting (behaviour), feedback on behaviour, self-monitoring of behaviour, and social support (unspecified). Two studies included a direct comparison of an intervention with and without a contract; the BCTs used in Franzini and Grimes’s study [42] (see Appendix 2) included the same common BCTs as the above studies, with the exception of 3.1 Social support. The individual BCTs in Nyer and Dellande’s [39] study were unavailable.

Given the focus of the remaining four studies [43–46] was on the delivery of the contracts, there were few differences within studies between the BCTs delivered to intervention and control groups. However, the common BCTs across these studies were similar to above: goal setting (behaviour), goal setting (outcome), self-monitoring of behaviour, feedback on outcome of behaviour, and social support (unspecified).

#### Intervention deliverer

Education level of the intervention deliverer was not reported in one study [44]. Of the remaining nine studies, all interventions were delivered by doctorate level or medically trained individuals or health care professionals, except for one, where the deliverers had minimum high school education and received 2 days training [40]. As the intervention in Kegler et al’s [41] study was effective, level of provider education does not seem to have affected outcomes.

#### Intervention delivery

The interventions in two studies were delivered to individuals alongside web and/or mail support [40, 41] whereas the others were delivered in group settings. The weight outcomes of the groups in Craighead and Blum’s [45] study suggests the importance of delivering the contract alongside a group intervention. The reasons for this are unclear, for example, the group setting may have introduced a form of social support, or social pressure, which may have enhanced the effect of the contract. However, given that the two interventions delivered individually were effective, it is possible that it is not the group setting itself that is important, but the ongoing contact, regardless of whether this is with a health professional or peers.

#### Intervention length

Of those studies comparing a commitment device intervention with no intervention, four studies delivered the intervention over a minimum of 16 weeks [38–41], whereas one was delivered over 11 weeks [42]. Given that there was no significant difference between the contract and no contract groups in this study, the length of intervention may be important, again suggesting the importance of ongoing support alongside contract delivery.

### Quality of studies

As seen in Table 3, quality varied across studies. Four trials (40%) described a true randomization procedure, and four (40%) stated participants were randomised but without describing the method used. The remaining two (22%) studies were identified as having a high risk of bias in relation to sequence generation. Allocation concealment was rated as unclear across seven (78%) studies; one was rated as high risk, with the remaining two (22%) studies identified as low risk of bias. Two (22%) studies described adequate blinding procedures, three did not, and five (56%) reported insufficient information regarding their blinding procedures. Other bias was rated as high across six (60%) studies. As can be seen in the table, the bias was rated as ‘unclear’ across several points and could not be determined, which reflects the poor reporting of studies in the aged literature.

**Table 3:** Bias ratings across all studies

	Sequence generation	Allocation concealment	Blinding	Incomplete outcome^a^	Incomplete outcome^b^	Selective reporting	Other bias
Balk-Møller	+	–	–	?	–	?	–
Black	?	?	?	+	+	+	–
Clifford	–	?	?	–	–	–	–
Craighead	?	?	?	n/a	?	+	–
Dubbert	?	?	?	+	+	+	+
Franzini	–	?	–	–	n/a	?	?
Kegler	+	+	+	+	+	?	+
Maruyama	+	?	+	n/a	–	+	–
Nyer	?	+	–	–	–	?	+
Ureda	+	?	?	?	?	?	–

^a^Short term data (2–6 weeks), ^b^Longer term data (> 6 weeks), − High risk of bias, + Low risk of bias,? Unclear

## Discussion

The results suggest that commitment devices may improve short and longer term weight loss outcomes and dietary change when delivered alongside an educational weight loss programme, although evidence for longer term maintenance of such behaviours is based on only two studies. Commitment devices in the included studies were all written (handwritten or online), and were mostly in the form of behavioural contracts, though there were promising results from one study for the use of public commitments to weight loss outcomes. No evidence was identified for the use of verbal commitments. These findings suggest that behavioural contracts may be useful to health professionals in facilitating dietary change among patients seeking weight loss support, and may be useful for those who would benefit from an improved diet e.g. people with diabetes or high cholesterol.

Commitment devices were effective with individually delivered interventions and in group settings, but not alongside minimal contact, suggesting that on-going contact with either health professionals or peers is needed. An important delivery element for behavioural contracts in particular is who co-signs or witnesses the contract: peers or intervention deliverers had the best effect on outcomes. All interventions covered both dietary and physical activity behaviours, and the BCTs used most commonly alongside successful commitment interventions were goal setting (1.1 behaviour & 1.3 outcome), feedback on behaviour (2.2), self-monitoring of behaviour (2.3), feedback on outcome of behaviour (2.7), and social support (unspecified, 3.1). The professional background of the intervention provider did not affect outcomes, as long as they had received adequate training.

Behavioural contracts were ineffective in increasing adherence to physical activity goals in the short or long-term. It is unclear why contracts increased adherence to dietary changes but did not promote physical activity. Dietary goals specified in the contracts may have been more acceptable to participants or easier to implement than the physical activity goals, which would suggest that more work is needed in relation to goal development. Previous work has identified the importance of autonomy/ autonomous motivation on physical activity behavioural outcomes in the context of Self-Determination Theory (SDT) [47–49]. This may explain the lack of effect on physical outcomes, as intrinsic motives for physical activity may have been undermined by the behavioural contract, which may be viewed as a form of extrinsic motivation within SDT, even without the financial incentive. Though this is contrary to findings around extrinsic and intrinsic motivation in relation to weight loss discussed earlier, that particular research included financial incentives attached to achieving weight loss goals, rather than specifically increasing physical activity levels [25]. Another explanation may be that changes in diet can have faster results in terms of weight loss when compared to increased exercise, and therefore people tend to focus on changing their diet alone. This is suggested by authors of a recent review of weight loss intervention adherence, which also found better adherence to dietary than physical activity, though they also suggest that this may be due to the nature of data collection which is usually reliant on self-report and recall which is subject to errors [50]. This would suggest that this phenomenon is relevant to all weight loss interventions, not just those including contracts.

The results found regarding the effect of publicity of commitment devices is in line with previous research which identifies that in order to be effective, commitments should be public, or have the ability to be made public, and where possible, should be made in groups [51–53]. Others have suggested that making commitments public maximises their effect by adding a form of social consequence [17]. The results from a recent review of goal setting across all behaviours suggest that goal setting was more effective when made publicly than when not made publicly [53]. In the same review, goal setting alongside a personal commitment made in private was less effective than goal setting alone, and the addition of a behavioural contract to goal setting made no difference to its effectiveness. However, the authors did not perform a meta-regression to take into account other variations, such as study design, population, design and setting. Epton and colleagues [53] highlight that they did not identify any studies targeting low socio-economic populations, who we feel would benefit from these soft commitment devices.

Previous research has identified that to maximise their effect, commitment devices such as behavioural contracts should be used alongside other BCTs [51, 52]. Previous reviews have identified that the inclusion of goal setting, self-monitoring of behaviour and social support as part of lifestyle interventions are associated with improved outcomes [54, 55], all of which were identified in this review. It is noteworthy that the BCT taxonomy (v.1) [30] stipulates that the BCTs behavioural contracts and commitments (both of which are types of commitment devices) must also be coded as goal setting (behaviour) when coding intervention components, and as such this common BCT was expected. The BCTs goal setting (outcome), self-monitoring of behaviour are also expected given the nature of weight loss interventions. However the BCT social support was not necessarily expected, and may be important to include alongside the delivery of contracts because they provide additional publicity of the contract. Indeed, a recent meta-analysis of weight loss intervention adherence reported that social support improved adherence rates by 29% [50].

Though studies referred to the development of the interventions as a whole, none discussed the development of the commitment device elements of the intervention in terms of importance and acceptability to those committing to them. This suggests more research is needed in relation to the development of these components. For example, though publicity of contracts may improve outcomes, the study that reported this [39] excluded those who didn’t consent to the public aspect of the contract, making the acceptability and feasibility of this unclear.

Though our limited results look promising, results must be interpreted with caution given the complexity and heterogeneity of the interventions, and the identified level of bias of studies included in this review. We have identified several limitations, including uncertainty in our estimate of mean weight loss; confidence intervals are wide and don’t rule out the possibility of minimal or zero effect in the longer term. Some trials did not report outcome data in sufficient detail to allow meta-analysis, therefore we cannot rule out bias due to selective outcome reporting. Given the limited number of trials identified, we were also unable to explore whether the effect of commitment device interventions is consistent across different settings, populations and genders. For example, we identified that only 14% of total participants included in the studies in this review were male, which is consistent with previous findings that males are underrepresented in weight loss interventions trials [56]. There has been a push in recent years to tailor interventions specifically for males, something which has been done successfully by designing an intervention around football training [57]. Identifying ways to encourage male participation in lifestyle interventions should be considered in future designs of such trials.

Given the length of time over which commitments have been used both within research and in the ‘real world’, the literature around commitment devices has not progressed as one would have expected; only two of the ten included studies compared the same intervention with and without a behavioural contract. As neither of these reported actual weight loss, or any dietary or physical activity outcomes, we were unable to assess the independent effect of behavioural contracts. In all cases, the commitment devices were combined with other BCTs, the majority of which were congruent with self-regulation theory. It was therefore difficult to observe the unique influence of commitment devices on weight and/or behavioural outcomes independent of other BCTs.

One important advancement in intervention development and evaluations in recent years is the development of a BCT taxonomy [30] to improve the reporting of intervention BCTs and allow replication. However, given that this is a relatively new advancement and much of the literature was conducted before it, few had reported their interventions with enough information to be confident that we were able to identify all BCTs. Indeed, only one of the full texts screened had described their intervention components using a taxonomy. Despite our best efforts, this poor reporting may also have limited the number of studies in this review, given that many commitment devices such as commitments and behavioural contracts are not always described as such.

This review is the first to exclude studies with financial rewards or incentives attached to the commitment device (e.g. hard commitments), and therefore focuses on the effect of the commitment itself rather than the financial motivators which have been identified elsewhere [19]. Previous reviews have suggested that contracts work best in the long-term where no financial incentives are included [21], but given that few studies in this review reported long-term outcomes, this review cannot provide evidence for this.

The need for the use of consistent outcome measures was highlighted in this review, such as BMI or actual weight loss (kg). Indeed, the COMET (Core Outcome Measures in Effectiveness Trials) initiative [58] is addressing this issue by developing an agreed set of standardised outcomes, with the aim of clearly identifying the outcomes that clinical trials should report in relation to specific conditions, in this case, obesity. Attention should be given to outcomes which will be of most use for implementation by health services in future research.

## Conclusion

Though the evidence is limited, the findings suggest that soft commitment devices, specifically non-financial behavioural contracts, have potential for facilitating short-term, and possibly longer term weight loss and dietary behaviour change when used alongside a lifestyle intervention. Including a public element to the contract, specifically where witnessed by a provider or peers, appear to improve outcomes. Behavioural contracts are relatively simple and brief to deliver and could be easily embedded into public health interventions, and their non-financial nature means that they would be suitable for all populations. However, our review identified no high quality direct comparisons of a weight management intervention with and without a commitment; this suggests there is a need for a high quality trial to determine if non-financial behavioural contracts significantly improve outcomes, independent of other BCTs, or with which BCTs they are most effective. Future work should also identify the most important and acceptable delivery components. Care should be taken in deciding on outcome variables in such a trial (weight, behaviour), and given the importance of weight loss maintenance, longer term outcomes should be included.

## Data Availability

This review was undertaken using the process outlined in the PRISMA guidelines. The protocol was registered online with PROSPERO, ID number: CRD42018102506.

## Appendix 1

**Table 4:** Medical Subject Heading (MeSH), Suggest Subject Terms (SST) and keywords used in search

Term Type		Terms
1.	MeSH	Obesity OR Overweight OR Obesity, Morbid OR Waist-Hip Ratio OR Waist Circumference OR Body Mass Index
	SST	Obesity OR Obesity, Morbid OR Waist-Hip Ratio OR Waist Circumference OR Body Mass Index
	Text	Overweight OR Obes* OR (BMI Or Body Mass Index)
2	MeSH	Weight loss
	SST	Weight loss OR Weight control
	Text	Weight adj2 loss OR Weight adj2Control OR Weight adj2 reduction OR Weight adj2 outcomes OR BMI adj2 reduction OR BMI adj2 decrease OR waist adj2 decrease OR waist adj2 reduction.
3	MeSH	Health Promotion OR Health Behaviour OR Life Style OR Diet OR Diet, Reducing OR Diet, Fat-Reducing OR Weight Reduction Programs OR Exercise OR Sports
	SST	Health Promotion OR Life Style OR Behavior OR Diet OR Diet, Reducing OR Restricted Diet OR Weight Reduction Programs OR Exercise OR Sports OR Physical activity
	Text	Health adj2 Behavio* OR Behavio* adj2 change* OR Lifestyle OR Lifestyle adj2 Change OR Exercise OR Walk* OR Jog*or Run* OR Cycl* OR Swim* OR Exercise, Aerobic OR Exercise, Physical OR Physical adj2 Activity OR Diet
4	MeSH	Motivation
	SST	Goals Setting OR Behavioral Objectives OR Behavior Contracting
	Text	Commit* OR Pledg* OR Behavio* NEAR Contract* OR Contingen* adj2 contract* OR Contract* OR Implementation adj2 Intention* OR Action adj2 planning OR Goal adj2 setting

## Appendix 2

**Table 5:** Details of content and delivery of commitment devices

Study ID	Commitment type & additional intervention details	Co-BCTs^a^	Witness	Renewed	Outcomes
Balk-Møller 2017 [38]	Commitment device- Pledge (online/app commitment) 1 at baseline, Pledge 2 at 22 weeks. Weight loss subgroup chose one of: lose weight, eat healthier, improve physical fitness, stronger body, maintain healthy lifestyle. Intervention content-self-reporting of diet and exercise, personalized feedback, suggestions for activities and programs, practical tips.	Intervention group:1.1/1.3, 1.9, 2.2, 2.3, 2.4, 2.5, 3.1, 4.1, 10.1, 10.3. Control: None	Online/app	No	Weight (O), waist circumference (O), % body fat body composition monitor (O).
Black 1983 [43]	Commitment device- Behavioural contract. Contract to record food, non-routine PA for 2 weeks in first session. Four contracts used: 1) altering eating habits 2) increasing activity 3) combined contract 4) maintenance or continuation of weight loss Intervention content- Limited information provided. Adapted from Stuart & Davis ‘slim chance in a fat world’ (1972) - no further information available.	All groups: 1.1, 1.8, 2.,3 2.6 Husband Contracting group: as above plus 3.1	Spouse or health professional, dependant on group assignment	Yes	Weight (O)
Clifford 1991 [36]	Commitment device - Behavioural contract. Individual behavioural health self-contracts, no further info. Intervention content- A self-directed change (SDC) intervention that applied cognitive-behavioural techniques to exercise adherence, weight reduction/maintenance, and stress management.	Intervention groups: 1.8, 1.1, 3.1, 1.2, Control group: None	Not specified	Yes	Weight (O), body composition (fat) (O) exercise adherence questionnaire (SR) and Cardiovascular fitness (oxygen consumption levels) (O)
Craighead 1989 [45]	Commitment device - Behavioural contract. Signed an exercise contract to complete at least 90 min of any exercise of their choice per week. Intervention content- ‘standard behavioural weight control procedures as described in Ferguson (1975) and Mahoney & Mahoney (1976).’ No further information available.	Intervention group: 1.1, 1.8, 2.2, 2.3, 2.5, 2.6, 4.1, 10.4. Contract only group: 1.1, 1.8, 2.2, 2.5, 4.1.	Not specified	No	Weight (O), Body fat (O), Fitness (step) test (O), Eating inventory questionnaire (SR)
Dubbert 1984 [46]	Commitment device - Behavioural contract, commitment. Contract/commitments by male spouse of female participants to make one or two changes to facilitate efforts Intervention content- ‘4-week educational phase about weight reduction, nutrition, techniques for controlling eating and safely increasing exercise, coping with negative emotions and self-defeating cognition, asserting oneself to obtain the necessary support from significant others, and the importance of keeping records and setting goals … All advised to cut back on calorie intake while increasing daily activity.’	All groups: 1.1, 1.2, 1.3, 2.2, 2.3, 2.4, 2.7, 3.1. Couples group: As above, plus 1.8, 1.9, 10.4.	Not specified	Unclear	Weight (SR)
Franzini 1980 [42]	Commitment device - Behavioural contract 3 contracts 1) membership contract 2) self-contract 3) significant person contract. Intervention content- Standard behavioural modification treatment package (Stuart 1971), which includes increasing aerobic walking and monitoring and improving dietary habits.	Intervention group: 2.2, 2.3, 4.2,5.1, 10.3, 10.4, 10.6. Contract group: As above, plus 1.1, 1.3, 1.8. Control group: None	Not specified	Yes	Weight (O), % body fat (skinfold test) (O)
Kegler 2016 [41]	Commitment device - Behavioural contract Healthy actions contract, building from 2 to 6 actions over 16 weeks. Intervention content- Social–cognitive theory informed intervention targeting home food and activity environments to reduce energy intake and increase physical activity, included a tailored home environment profile, goal setting.	Intervention groups: 1.1, 1.8, 12.1, 12.6. Control group: 1.1, 2.3, 5.1, 12.6	Health coach	Yes	Weight (O), Energy intake (recall questionnaire) (SR), Physical Activity (Accelerometer) (O)
Maruyama 2010 [40]	Commitment device - Behavioural contract, consisting of a commitment sheet- dietary (food groups) and physical activity (steps). Intervention content- Monthly contact with trained dietician and physical trainer, including goal setting and action planning sessions and self-reporting diet & physical activity, followed by progression to online support.	Intervention group: 1.1, 1.8, 1.4, 2.3, 2.2, 3.1, Control group: None	Not specified	Yes	Dietary adherence (SR), PA adherence – pedometer (O), Weight / BMI (O)
Nyer 2010 [38]	Commitment device - Public commitment (outcome) 1) Weight loss goal and name displayed in glass cabinet for 16 weeks. 2) As above, but for 3 weeks only. 3) No public commitment. Intervention content- weight loss program ‘focus was on helping clients to transform their lives by identifying sustainable means of increasing caloric expenditure while reducing caloric input within the context of their lives.’ No further information.	Intervention groups: 1.3, 2.5 Control group: 2.5	Staff /peers at health centre (public)	No	Weight – not reported (O) % weight loss goal achieved (O)
Ureda 1980 [44]	Commitment device - Behavioural contracts. Three contracts: 1) Program participation, 2) completion of a nutrition education workbook, 3) establishment of weight-controlling regime. Intervention content- ‘behavioural self-management/self-help weight control program, whereby each participant was taught the skills and knowledge necessary to formulate and implement an individually tailored diet, meal pattern, and exercise regimen sufficient to achieve a weight loss of one-to-three pounds per week.’	All groups: 1.8, 1.1, 10.7	None or peer/ relative/ friend dependant on group	No	Weight (O)

^a^1.1 Goal setting (behaviour), 1.2 Problem solving, 1.3 Goal setting (outcome), 1.4 Action planning, 1.8 Behavioural contract, 1.9 Commitment, 2.2 Feedback on behaviour, 2.3 Self-monitoring of behaviour, 2.4 Self-monitoring of outcome(s) of behaviour, 2.5 Monitoring outcome(s) of behaviour by others without feedback, 2.6 Biofeedback, 2.7 Feedback on outcome(s) of behaviour, 3.1 Social support (unspecified), 4.1 Instruction on how to perform a behaviour, 5.1 Information about health consequences, 10.3 Non-specific reward, 10.4 Social reward, 10.6 Non-specific incentive, 10.7 Self-incentive, 12.1 Restructuring the physical environment, 12.5 Adding objects to the environment, 12.6 Body changes (Michie et al., 2013)[30]

*O* Objective measure, *SR* self-report measure

## Abbreviations

BCT: Behaviour change technique
BCTTv1: Behaviour change technique taxonomy version 1
BMI: Body mass index

## References

[1] World Health Organisation, Global Health Observatory data, 2015 www.who.int/gho/ncd/risk_factors/overweight/en/. Accessed 5 June 2019.

[2] Kodama S, Horikawa C, Fujihara K, Yoshizawa S, Yachi Y, Tanaka S, Ohara S, Matsunaga S, Yamada T, Hanyu O (2014). Quantitative relationship between body weight gain in adulthood and incident type 2 diabetes: a meta-analysis. Obes Rev.

[3] Suk SH, Sacco RL, Boden-Albala B, Cheun JF, Pittman JG, Elkind MS, Paik MC (2003). Abdominal obesity and risk of ischemic stroke the northern Manhattan stroke study. Stroke.

[4] Nakamura K, Fuster JJ, Walsh K (2014). Adipokines: a link between obesity and cardiovascular disease. J Cardiol.

[5] Moghaddam AA, Woodward M, Huxley R (2007). Obesity and risk of colorectal Cancer: a meta-analysis of 31 studies with 70,000 events. Cancer Epidemiol Biomark Prev.

[6] Flegal KM, Kit BK, Orpana H, Graubard BI (2013). Association of all-cause mortality with overweight and obesity using standard body mass index categories: a systematic review and meta-analysis. JAMA.

[7] Lehnert T, Sonntag D, Konnopka A, Riedel-Heller S, König H-H (2013). Economic costs of overweight and obesity. Best Pract Res Clin Endocrinol Metab.

[8] Dobbs R, Sawers C, Thompson F, et al. Overcoming obesity: An initial economic analysis. McKinsey Global Institute, 2014. http://www.mckinsey.com/insights/economic_studies/how_the_world_could_better_fight_obesity. Accessed 20 June 2019.

[9] Poobalan AS, Aucott LS, Smith WCS, Avenell A, Jung R, Broom J (2007). Long-term weight loss effects on all cause mortality in overweight/obese populations. Obes Rev.

[10] Wing RR, Lang W, Wadden TA, Safford M, Knowler WC, Bertoni AG, Hill JO, Brancati FL, Peters A, Wagenknecht L (2011). Benefits of modest weight loss in improving cardiovascular risk factors in overweight and obese individuals with type 2 diabetes. Diabetes Care.

[11] Orchard M, Fowler S, Temprosa M (2005). Impact of intensive lifestyle and metformin therapy on cardiovascular disease risk factors in the diabetes prevention program. Diabetes Care.

[12] Franz MJ, VanWormer JJ, Crain AL, Boucher JL, Histon T, Caplan W, Bowman JD, Pronk NP (2007). Weight-loss outcomes: a systematic review and meta-analysis of weight-loss clinical trials with a minimum 1-year follow-up. J Am Diet Assoc.

[13] Swift DL, Johannsen NM, Lavie CJ, Earnest CP, Church TS (2014). The role of exercise and physical activity in weight loss and maintenance. Prog Cardiovasc Dis.

[14] Heymsfield SB, Harp JB, Reitman ML, Beetsch JW, Schoeller DW, Erondu N, Pietrobelli A (2007). Why do obese patients not lose more weight when treated with low-calorie diets? A mechanistic perspective. Am J Clin Nutr.

[15] Alhassan S, Kim S, Bersamin A, King AC, Gardner CD (2008). Dietary adherence and weight loss success among overweight women: results from the a TO Z weight loss study. Int J Obes.

[16] Bryan G, Karlan D, Nelson S (2010). S. *Commitment devices*. Annu. Rev Econ.

[17] Service O, Hallsworth M, Halpbern D, Algate F, Gallagher R, Nguyen S, Ruda S, and Sanders M. EAST: Four simple ways to apply behavioural insights, in Behavioural Insight Team, London*.* 2014. https://www.behaviouralinsights.co.uk/wp-content/uploads/2015/07/BIT-Publication-EAST_FA_WEB.pdf. Accessed 10 Feb 2018.

[18] Bosch-Capblanch X, Abba K, Prictor M, Garner P. Contracts between patients and healthcare practitioners for improving patients' adherence to treatment, prevention and health promotion activities. Cochrane Libr. 2007. 10.1002/14651858.CD004808.pub3. 10.1002/14651858.CD004808.pub3 PMC6464838 17443556

[19] Sykes-Muskett BJ, Prestwich A, Lawton RJ, Armitage CJ (2015). The utility of monetary contingency contracts for weight loss: a systematic review and meta-analysis. Health Psychol Rev.

[20] Ananthapavan J., Peterson A., Sacks G. (2017). Paying people to lose weight: the effectiveness of financial incentives provided by health insurers for the prevention and management of overweight and obesity - a systematic review. Obesity Reviews.

[21] Lokhorst AM, Werner C, Staats H, van Dijk E, Gale JL (2013). Commitment and behavior change: a meta-analysis and critical review of commitment-making strategies in environmental research. Environ Behav.

[22] McGuire MT, Wing RR, Klem ML, Lang W, Hill JO (1999). What predicts weight regain in a group of successful weight losers?. J Consult Clin Psychol.

[23] Deci EL, Koestner R, Ryan RM (1999). A meta-analytic review of experiments examining the effects of extrinsic rewards on intrinsic motivation. Psychol Bull.

[24] Cerasoli CP, Nicklin JM, Ford MT (2014). Intrinsic motivation and extrinsic incentives jointly predict performance: a 40-year meta-analysis. Psychol Bull.

[25] Sen AP, Huffman D, Loewenstein G, Asch DA, Kullgren JT, Volpp KG, Cohen G, Fernandez Lynch H, Robinson CT (2017). Do financial incentives reduce intrinsic motivation for weight loss? Evidence from two tests of crowding out. Nudging health: health law and behavioral economics.

[26] Giles EL, Holmes M, McColl E, Sniehotta FF, Adams JM (2015). Acceptability of financial incentives for breastfeeding: thematic analysis of readers’ comments to UK online news reports. BMC Pregnancy Childbirth.

[27] Blaga Oana M, Vasilescu Livia, Chereches Razvan M (2017). Use and effectiveness of behavioural economics in interventions for lifestyle risk factors of non-communicable diseases: a systematic review with policy implications. Perspectives in Public Health.

[28] Lesser LI, Thompson CA, Luft HS (2018). Association between monetary deposits and weight loss in online commitment contracts. Am J Health Promot.

[29] NICE; National Institute for Health and Care Excellence, Obesity: identification, assessment and management of overweight and obesity in children, young people and adults. NICE clinical guideline 189*.*, 2014: guidance.nice.org.uk/cg189. 25535639

[30] Michie S, Richardson M, Johnston M, Abraham C, Francis J, Hardeman W, Eccles MP, Cane J, Wood CE (2013). The behavior change technique taxonomy (v1) of 93 hierarchically clustered techniques: building an international consensus for the reporting of behavior change interventions. Ann Behav Med.

[31] Barte Jeroen C. M., Wendel-Vos G. C. Wanda (2015). A Systematic Review of Financial Incentives for Physical Activity: The Effects on Physical Activity and Related Outcomes. Behavioral Medicine.

[32] ERC data collection form for intervention reviews for RCTs and non-RCTs. 2016. Available from: https://training.cochrane.org/interactivelearning/module-4-selecting-studies-and-collecting-data. Accessed 5 June 2019.

[33] Higgins JPT and Green S, Cochrane handbook for systematic reviews of interventions. Vol. 5. 2018. Available from: https://training.cochrane.org/handbook. Accessed 7 June 2019.

[34] Byrt T, Bishop J, Carlin JB (1993). Bias, prevalence and kappa. J Clin Epidemiol.

[35] Wood CE, Richardson M, Johnston M, Abraham C, Francis J, Hardeman W, Michie S (2015). Applying the behaviour change technique (BCT) taxonomy v1: a study of coder training. Transl Behav Med.

[36] Clifford PA, Tan SY, Gorsuch RL (1991). Efficacy of a self-directed behavioral health change program: weight, body composition, cardiovascular fitness, blood pressure, health risk, and psychosocial mediating variables. J Behav Med.

[37] Rodgers M, Sowden A, Petticrew M, Arai L, Roberts H, Britten N, Popay J (2009). Testing methodological guidance on the conduct of narrative synthesis in systematic reviews effectiveness of interventions to promote smoke alarm ownership and function. Evaluation.

[38] Balk-Møller Nina Charlotte, Poulsen Sanne Kellebjerg, Larsen Thomas Meinert (2017). Effect of a Nine-Month Web- and App-Based Workplace Intervention to Promote Healthy Lifestyle and Weight Loss for Employees in the Social Welfare and Health Care Sector: A Randomized Controlled Trial. Journal of Medical Internet Research.

[39] Nyer PU, Dellande S (2010). Public commitment as a motivator for weight loss. Psychol Mark.

[40] Maruyama C, Kimura M, Okumura H, Hayashi K, Arao T (2010). Effect of a worksite-based intervention program on metabolic parameters in middle-aged male white-collar workers: a randomized controlled trial. Prev Med.

[41] Kegler MC, Haardörfer R, Alcantara IC, Gazmararian JA, Veluswamy JK, Hodge TL, Addison AR, Hotz JA (2016). Impact of improving home environments on energy intake and physical activity: a randomized controlled trial. Am J Public Health.

[42] Franzini LR, Grimes WB (1980). Contracting and Stuart's three-dimensional program in behavior modification of the obese. Psychotherapy Theory Res Pract.

[43] Black DR, Lantz CE (1984). Spouse involvement and a possible long-term follow-up trap in weight loss. Behav Res Ther.

[44] Ureda JR (1980). The effect of contract witnessing on motivation and weight loss in a weight control program. Health Educ Q.

[45] Craighead LW, Blum MD (1989). Supervised exercise in behavioral treatment for moderate obesity. Behav Ther.

[46] Dubbert PM, Wilson G (1984). Goal-setting and spouse involvement in the treatment of obesity. Behav Res Ther.

[47] Ng JYY, Ntoumanis N, Thogersen-Ntoumani C, Deci EL, Ryan RM, Duda JL, Williams GC (2012). Self-determination theory applied to health contexts: a meta-analysis. Perspect Psychol Sci.

[48] Teixeira PJ, Carraça EV, Markland D, Silva MN, Ryan RM (2012). Exercise, physical activity, and self-determination theory: a systematic review. Int J Behav Nutr Phys Act.

[49] Teixeira PJ, Carraça EV, Marques MM, Rutter H, Oppert JM, De Bourdeaudhuij I, Lakerveld J, Brug J (2015). Successful behavior change in obesity interventions in adults: a systematic review of self-regulation mediators. BMC Med.

[50] Lemstra M, Bird Y, Nwankwo C, Rogers M, Moraros J (2016). Weight loss intervention adherence and factors promoting adherence: a meta-analysis. Patient Prefer Adherence.

[51] Cialdini RB (1993). Influence: science and practice.

[52] McKenzie-Mohr D, Smith W (2008). Fostering sustainable behaviour.

[53] Epton T, Currie S, Armitage CJ (2017). Unique effects of setting goals on behavior change: systematic review and meta-analysis. J Consult Clin Psychol.

[54] Dombrowski S, Sniehotta FF, Avenell A, Johnston M, MacLennan G, Araújo-Soares V (2010). Identifying active ingredients in complex behavioural interventions for obese adults with obesity-related co-morbidities or additional risk factors for co-morbidities: a systematic review. Health Psychol Rev.

[55] Greaves CJ, Sheppard KE, Abraham C, Hardeman W, Roden M, Evans PH, Schwarz P (2011). Systematic review of reviews of intervention components associated with increased effectiveness in dietary and physical activity interventions. BMC Public Health.

[56] Pagoto SL, Schneider KL, Oleski JL, Luciani JM, Bodenlos JS, Whited MC (2012). Male inclusion in randomized controlled trials of lifestyle weight loss interventions. Obesity.

[57] Hunt K, Wyke S, Gray CM, Anderson AS, Brady A, Bunn C, Donnan PT, Fenwick E, Grieve E, Leishman J, Miller E, Mutrie N, Rauchhaus P, White A, Treweek S (2014). A gender-sensitised weight loss and healthy living programme for overweight and obese men delivered by Scottish premier league football clubs (FFIT): a pragmatic randomised controlled trial. Lancet.

[58] Prinsen CAC, Vohra S, Rose MR, King-Jones S, Ishaque S, Bhaloo Z, Adams D, Terwee C (2014). Core outcome measures in effectiveness trials (COMET) initiative: protocol for an international Delphi study to achieve consensus on how to select outcome measurement instruments for outcomes included in a ‘core outcome set. Trials.

